# Investigations on the Nonlinear Optical Properties of 0D, 1D, and 2D Boron Nitride Nanomaterials in the Visible Spectral Region

**DOI:** 10.3390/nano13121849

**Published:** 2023-06-13

**Authors:** Stefanie Dengler, Bernd Eberle

**Affiliations:** Fraunhofer IOSB, Fraunhofer Institute of Optronics, System Technologies and Image Exploitation, 76275 Ettlingen, Germany; bernd.eberle@iosb.fraunhofer.de

**Keywords:** optical limiting, nonlinear absorption, nonlinear scattering, boron nitride nanomaterials

## Abstract

In recent years, boron nitride nanomaterials have attracted increasing attention due to their unique properties such as high temperature stability and high thermal conductivity. They are structurally analogous to carbon nanomaterials and can also be generated as zero-dimensional nanoparticles and fullerenes, one-dimensional nanotubes and nanoribbons, and two-dimensional nanosheets or platelets. In contrast to carbon-based nanomaterials, which have been extensively studied during recent years, the optical limiting properties of boron nitride nanomaterials have hardly been analysed so far. This work summarises a comprehensive study on the nonlinear optical response of dispersed boron nitride nanotubes, boron nitride nanoplatelets, and boron nitride nanoparticles using nanosecond laser pulses at 532 nm. Their optical limiting behaviour is characterised by means of nonlinear transmittance and scattered energy measurements and a beam profiling camera is used to analyse the beam characteristics of the transmitted laser radiation. Our results show that nonlinear scattering dominates the OL performance of all measured boron nitride nanomaterials. Boron nitride nanotubes show a large optical limiting effect, much stronger than the benchmark material, multi-walled carbon nanotubes, which makes them promising for laser protection applications.

## 1. Introduction

A scientific breakthrough with a revolutionary impact on daily life happens very rarely. The laser is one of those inventions, which, in fact, has changed the world. People said it was just a new light source without any application—how different the situation is nowadays. Whether in optical communication, in printing, in scanners, for material processing, or for operations, it is impossible to imagine our everyday life, industry, and medicine without it—and the potential of the laser is far from being exhausted.

On the other hand, laser radiation poses a serious and growing threat to sensors and the human eye. The broad and growing range of applications is leading to an increasing use of lasers in all areas of life. In addition to lasers with large output power, more and more different laser wavelengths are available. As a result, the need to protect sensors and the human eye from laser-induced damage is also constantly increasing. Since effective protection must be directed against all possible expected wavelengths, eye or sensor protection with conventional optical means (e.g., with absorption or interference filters) is no longer effective. New concepts are required that attenuate laser radiation to a tolerable intensity, independent of a specific wavelength.

**Figure 1:**
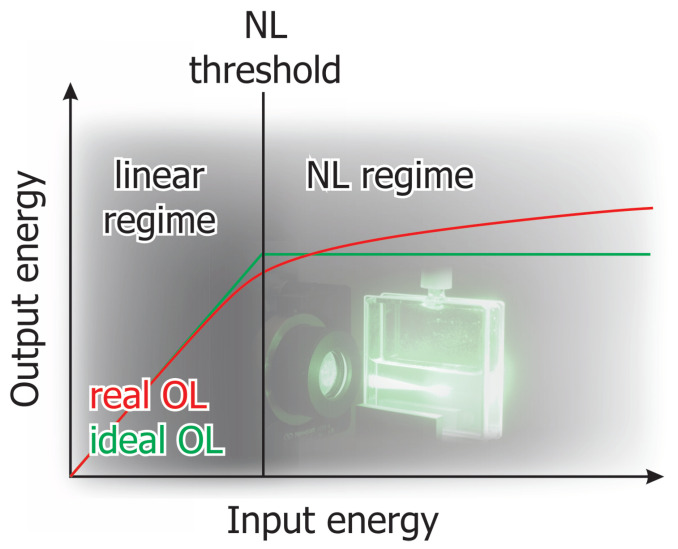
Schematic drawing illustrating ideal and real OL performance.

A possible protection concept can be realised using nonlinear optical (NLO) materials. Optical limiting (OL) is an NLO phenomenon where the properties of a material change in such a way that it leads to an intensity-dependent attenuation of laser radiation. Thus, the transmitted energy is only slightly dependent on increasing input energy. A linear relationship between input and output energy only exists for small input energies. The behaviours of ideal and real OL are shown schematically in Figure 1.

A variety of NLO effects that can trigger OL behaviour are described in the literature and can be assigned to three mechanisms: nonlinear (NL) absorption, NL scattering, and NL refraction [1,2,3,4]. A matching optical limiter can effectively attenuate a potentially dangerous laser beam to a level human eyes or optical sensors can be exposed to without permanent damage. With regards to eye protection, OL materials that exhibit NL attenuation for pulsed laser radiation in the visible (VIS) spectral range are of particular interest, as long as near infrared radiation is sufficiently blocked. A large OL effect has already been found in metallic and organometallic compounds [5,6,7], carbon-based nanomaterials [8,9,10,11,12], inorganic nanoparticles [13,14,15], and their hybrid materials [16,17,18]. In recent years, two-dimensional layered nanomaterials have received significant attention due to their unique optical properties and potential applications in various fields, including OL. Various two-dimensional materials, such as graphene and graphene oxide, but also the transition metal dichalcogenides, MXene and black phosphorus, exhibit promising OL properties [19,20,21,22]. However, up to now, no completely satisfactory material for OL applications is known that fulfils the requirements for eye protection, such as a low threshold, sufficient attenuation, and broadband efficiency. For this reason, research continues into new materials with better OL efficiency and attempts to improve the properties of known materials.

Boron nitride (BN) nanomaterials are promising inorganic nanosystems with unique properties such as high temperature stability and high thermal conductivity [23,24]. BN is a compound consisting of equal numbers of alternatively linked boron and nitrogen atoms. Depending on temperature and pressure it can crystallise in four different forms [25,26]: hexagonal, rhombohedral, cubic, and wurtzite, with hexagonal boron nitride as the most common form since it is the most stable at room temperature. Like carbon nanomaterials, they can be divided into zero-dimensional nanoparticles and fullerenes, one-dimensional nanotubes and nanoribbons, and two-dimensional nanosheets or nanoplatelets. They show a large number of potential applications in electronic and biomedical applications [27,28]. However, their NLO properties have hardly been investigated so far.

This work summarises a comprehensive study on the NLO response of dispersed BN nanotubes (BNNT), BN nanoplatelets (BNNS), and BN nanoparticles (BNNP) using nanosecond laser pulses at 532 nm. To evaluate their properties, a comparative analysis using multi-walled carbon nanotubes (MWCNT), one of the carbon-based benchmark materials, is performed. In order to investigate the beam characteristics of the transmitted laser radiation, a beam profiling camera was used. Usually, in studies of OL properties only the transmitted energy is measured. Thus, there is generally no information about the beam characteristics and the energy distribution of the transmitted laser beam. To our best knowledge, the NLO characteristics of such boron nitride nanomaterials have never been measured and analysed in detail before.

## 2. Mechanisms for Optical Limiting

There are two ways to reduce the intensity of the impinging laser radiation when using OL devices for laser protection. Either a part of the laser beam is absorbed or it is distributed over a larger area. A variety of NLO effects can trigger OL behaviour, the most important effects are briefly presented in this section.

### 2.1. Nonlinear Scattering

**Figure 2:**
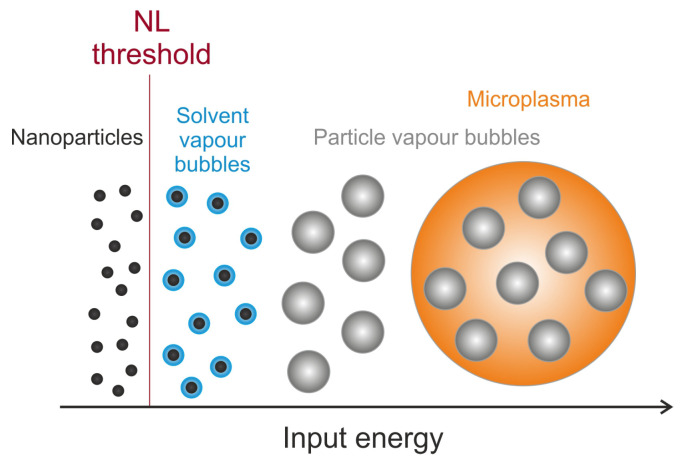
Schematic drawing of the different scattering centres with increasing irradiation.

NL scattering can disperse laser radiation into a larger solid angle, which reduces the resulting intensity of the incident beam. This effect has already been described for various nanomaterials [2]. However, the nanoparticles themselves are too small to form effective scattering centres. According to Mie theory [29], no relevant scattering can be achieved by particles much smaller than the wavelength of the incident radiation. Scattering becomes effective when further, significantly larger scattering centres are formed by interaction of the laser radiation with the nanoparticles. NL scattering is an induced process, since energy is deposited in the particles due to absorption. Thermal energy transfer to the surroundings leads to the formation of solvent bubbles and thus dynamically growing scattering centres around the nanoparticles. With increasing irradiation, fast growing scattering centres are generated by vapourisation and subsequent ionisation of the particles. The generation of solvent bubbles take place at lower energy densities, while the vapour bubbles and the microplasmas arise at higher energy densities (Figure 2).

Belousova et al. [30,31] developed a theoretical model to describe the OL properties of carbon nanoparticles at moderate laser intensities close to the onset of NL behaviour. The OL process is described in three steps: dynamics of formation and expansion of solvent vapour bubbles, Mie scattering of expanding bubbles, and NL propagation through the scattering medium. Belousova’s simulation indicates among other things, that the scattering cross-section rises significantly with increasing scattering centre radius. The scattering behaviour of CNT suspensions was also described using Mie theory, by Vivien et al. [32]. It applies here too, since the scattering centres in this case are also spherical bubbles.

### 2.2. Nonlinear Absorption

The absorption coefficient of NL absorption depends on the intensity, thus there is no linear relationship between the incident and transmitted intensity of the laser radiation. For OL applications, materials with steeply increasing absorption coefficients are suitable, whereby a large attenuation at high intensities is achieved. Typical mechanisms are multi-photon absorption (MPA), excited-state absorption (ESA), or reverse saturable absorption (RSA).

**Figure 3:**
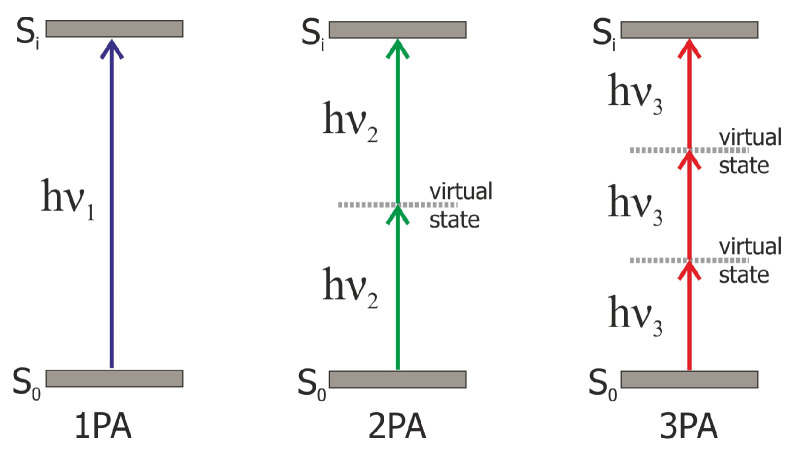
Energy level diagram of one-photon absorption (1PA), two-photon absorption (2PA), and three-photon absorption (3PA).

MPA [1,33,34] is an absorption process where an atom or molecule is excited from one state (generally the ground state) to an excited state by simultaneous absorption of two or more photons. The energy level diagrams of 1PA, 2PA, and 3PA is schematically shown in Figure 3. Many metallic and semiconductor nanomaterials, quantum dots, chromophores, and conjugated polymers possess MPA-induced OL [34].

**Figure 4:**
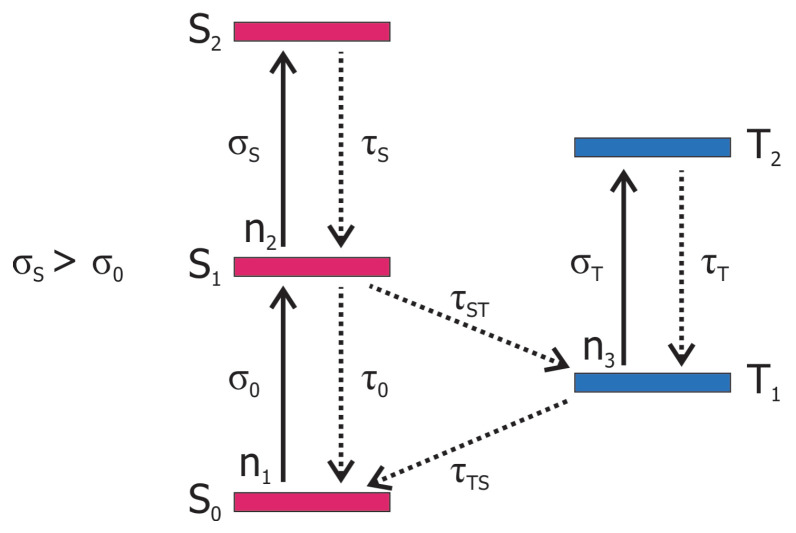
Five-level model of RSA. S${}_{i}$ represent singlet states, T${}_{i}$ triplet states, $\sigma_{i}$ absorption cross-sections, and $\tau_{i}$ relaxation times.

Materials which bleach under intense optical irradiation are called saturable absorbers, they are key elements in passive mode-locked and Q-switched lasers. The reverse effect of increasing absorption was reported for the first time by Giuliano and Hess, in 1967 [35], for the dyes sudanschwartz-B and indanthrone. The process of RSA [3,5,36] involves the multi-step absorption of photons with the same wavelength from the ground state and from excited states. This sequential multi-step process can be described by a five-level model (Figure 4); for simplicity, vibronic levels are neglected. Representative materials showing RSA are fullerenes, phtalocyanines, and porphyrines [1,3,5,6].

ESA usually becomes very important at high incident light intensities due to significant population of excited states. In molecular systems, a high density of states can be present close to the state involved in the excitation. An excited electron can rapidly undergo a transition to one of these states before it decays back to the ground state. There may also be a number of higher-lying excited states coupled to these intermediate excited states, with energy differences in near-resonance to the incident photon energy. Thus, the electron can be promoted into a higher-lying excited state due to absorption before it relaxes to the ground state [37,38]. In semiconductors, charge carriers can be generated in the conduction band by one-photon or two-photon excitation. With sufficiently high intensities, these free carriers can be excited to higher-lying states in the conduction or valence band by absorbing additional photons. This process is called free-carrier absorption (FCA), which is similar to ESA in molecular systems [1].

### 2.3. Nonlinear Refraction

The effect of an intensity-dependent refractive index change is called NL refraction [1,3,4,33]. A variety of physical processes, such as the optical Kerr effect or absorption-induced heating, can lead to an intensity-dependent refractive index. The intensity-dependent refractive index *n*(*I*) of an isotropic material can be calculated using the equation

(1) $$n\left(I\right)=n_{0}+n_{2}I,$$

taking into account NLO effects up to third order, where $n_{0}$ is the linear refractive index and $n_{2}$ the NL refractive index. In case of an optical Kerr effect [39,40], the refractive index change is caused by the electric field of the incident radiation. As with NL scattering, the transmitted laser radiation is dispersed into a larger solid angle; if an aperture is placed behind the sample, a relevant portion of the laser beam can be blocked.

The refractive index of a material can also change due to thermal processes [41]. If a part of the laser radiation is absorbed, the temperature in the irradiated area increases, which leads to a local change in density [42]. In a strongly focused laser beam, this thermally induced refractive index change builds up fast enough to effectively influence nanosecond laser pulses [43]. This is in fact the main effect of NL refraction for continuous wave and nanosecond pulsed laser radiation [44,45]. The thermal lens effect is a consequence of absorption and can hardly be avoided. Thermally induced refractive index changes can easily reach the order of $10^{-5}$ cm${}^{2}$/W, while $\chi^{\left(3\right)}$ refractive index changes are usually at least five orders of magnitude smaller [46].

## 3. Materials and Methods

### 3.1. Sample Preparation

BNNP (boron nitride nanopowder, <150 nm average particle size), BNNT (boron nitride nanotubes, multi-walled, powder, purity > 90%, diameter 30–50 nm, length > 10 µm), BNNS (boron nitride nanoplatelets, lateral dimensions < 5 µm), MWCNT (>98% carbon basis, O.D. × L 6–13 nm × 2.5–20 µm), and polyvinylpyrrolidone (PVP) (powder, average Mw ∼29,000) were purchased from Merck KGaA. Ethanol (ROTIPURAN^®^ ≥ 99.8%, p.a.), and nitric acid (65%, pure) were bought from Carl Roth GmbH + Co. KG., Karlsruhe, Germany.

Pristine BNNT are rarely soluble in organic and aqueous solutions since they form bundles due to van der Waals interactions during the synthesis [47]. To generate stable BNNT dispersions, different polymers such as PVP or poly(ethylene glycol) can be used [48]. To prepare stable BN dispersions, a PVP ethanol solution (1 mg/mL) was used, since PVP is not only suitable for BNNT, but also for BNNS and BNNP. The samples were sonicated in an ultrasonic bath for 3 h. MWCNT was treated according to the procedure described in a previous work [11]. With the help of an ultrasonic bath, a dispersion of MWCNT in deionised water was created.

Transmittance spectra of the samples were recorded on a Shimadzu UV-3600 UV–Vis–NIR spectrophotometer. A quartz cuvette with a 10 mm optical path length was used for all optical measurements. Scanning electron microscopy (SEM) characterisation was performed using a Hitachi SU-70 at 5 kV. The SEM samples were prepared by placing a droplet of the dispersion onto a silicon wafer and subsequently dried in air.

### 3.2. Experimental Setup

**Figure 5:**
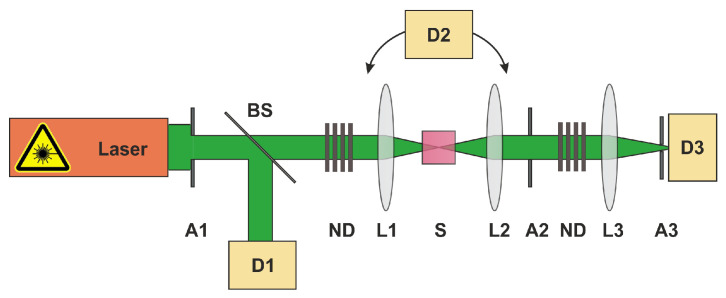
Experimental setup to study NL transmittance and scattering. With L1, L2, and L3—lenses; S—sample; D1 and D2—photodiodes; D3—photodiode/beam profiling camera; BS—beamsplitter; A1, A2, and A3—apertures.

The experimental setup to measure the NLO response (NL transmittance and scattering) is described in detail elsewhere [49]. A sketch of the setup used for the OL studies is shown in Figure 5. In brief, we used a frequency-double Nd:YAG laser system (Coherent Infinity) working at a wavelength of 532 nm at a 1 Hz pulse repetition rate with a pulse length of around 3 ns. The light emitted from the laser was chosen to be horizontally polarised (p-polarised). With the help of a beam splitter, the beam was divided into a reference and a signal beam. The reference beam was used to determine the energy of the laser pulses (detector D1). The samples were placed in the intermediate focal plane of a f/5 Keplerian telescope formed by the lenses L1 and L2 (focal lengths 60 mm and 100 mm). The diameter of the laser focus was measured by the knife-edge method to be 5 µm at 532 nm. The focusable energy (or encircled energy), which is generally defined as the energy detected within a solid angle of 1.5 mrad [4], was measured using lens L3 (focal length 400 mm) and pinhole A3 (diameter 600 µm) in front of the signal photodiode D3. This is often used to benchmark OL materials for laser protection applications. With the help of detector D2, the portion of scattered radiation at 30° in the forward direction was simultaneously recorded. To measure the angle-dependent scattering from 30° to 140°, the detection system D2 was mounted on a rotating stage. The sector from 30° to 90° represents forward scattering, whereas the sector from 90° to 140° corresponds to backscattering. In order to investigate the beam characteristics of the transmitted laser radiation, a beam profiling camera (WinCamD-LCM, Dataray) was positioned in the focal plane of lens L3 and the pinhole A3 was removed. All detectors (D1–D3) were equipped with bandpass filters (Edmund Optics, OD > 6, centre wavelength 532 nm, FWHM: 3.7 nm).

In studies of OL properties, only the transmitted energy is measured in general. Thus, there is usually no information about the beam characteristics and the energy distribution of the transmitted laser beam. However, for the evaluation of the OL efficiency, this information is important, since both the transmitted energy and its intensity distribution play a role in assessing the effectiveness of a material in laser protection applications.

## 4. Results and Discussion

### 4.1. Sample Characterisation

**Figure 6:**
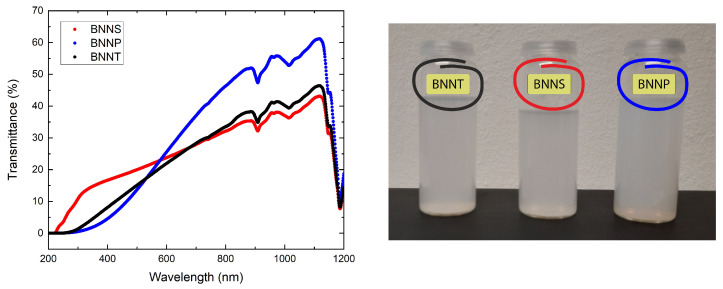
Transmittance spectra of the dispersions (BNNT, BNNS, and BNNP) in PVP ethanol solution from 200 nm to 1200 nm (**left**) and photograph of the samples (**right**).

In order to start from similar conditions, the concentrations of BNNT, BNNS, and BNNP were adjusted to have approximately the same transmittance around the used laser wavelength of 532 nm. The transmittance spectra (left) and a photograph of the samples (right) are shown in Figure 6.

**Figure 7:**
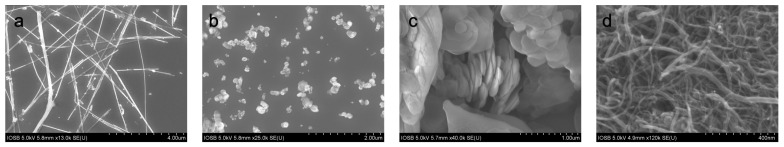
SEM micrographs of BNNT (**a**), BNNP (**b**), BNNS (**c**) and MWCNT (**d**).

To examine the morphological characteristics of the samples, SEM measurements were performed. The SEM micrographs of BNNT (a), BNNP (b), BNNS (c), and MWCNT (d) are shown in Figure 7. The BNNT sample (a) consists mainly of nanotubes, but there are also some nanoparticles of different sizes present. BNNP (b) are for the most part approximately spherical particles with sizes specified by the manufacturer of less than 150 nm. BNNS are platelets of different lateral dimensions (<5 µm), which can be clearly seen from the vertically positioned BNNS in the SEM micrograph (c). MWCNT (d) consists almost entirely of nanotubes, there are virtually no other structures, such as nanoparticles, present.

### 4.2. Nonlinear Optical Studies

The OL properties of the prepared BN dispersions were investigated using nanosecond laser pulses at 532 nm. In order to assess the potential of BN nanomaterials for OL applications, the results were compared with MWCNT. CNT (single- or multi-walled) dispersions are known to be suitable as optical limiters since their OL performance has been widely studied during recent years. It is generally believed that NL scattering dominates the NLO properties of CNT dispersions [9,32,50]. In the following, the dispersions of BNNT, BNNP, and BNNS in ethanol and PVP are referred to as BNNT, BNNP, and BNNS, respectively, the dispersion of MWCNT in H${}_{2}$O as MWCNT, for simplicity.

**Figure 8:**
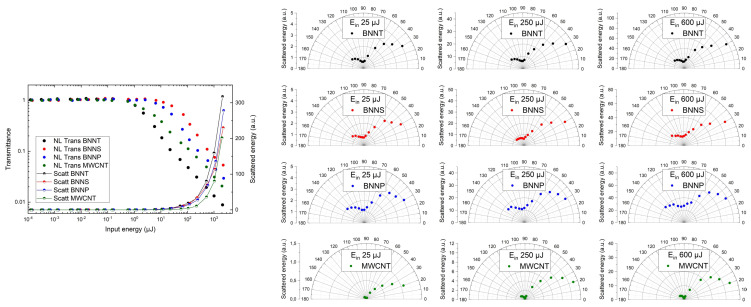
Normalised NL transmittance and scattering at 30° in the forward direction as a function of the incident pulse energy (left) and polar scattering diagrams from 30° to 140° at three different input energies (E_in_) of BNNT, BNNS, BNNP, and MWCNT.

The normalised NL transmittance and NL scattering at 30° for our samples, plotted as a function of the incident pulse energy, is shown in Figure 8 on the left. The input energy was varied from around 0.1 nJ up to 2 mJ. At low input energies, the transmittance is constant and thus independent of the irradiated energy. With further increasing input energy, the transmittance becomes intensity-dependent and decreases. The transmittance curve can therefore be divided into a linear and, beyond the onset of NL behaviour, into an NL part, with intensity-dependent NL attenuation. The transmittance starts to decrease at around 0.5 μJ for BNNT and MWCNT and around 2.5 μJ for BNNS and BNNP. The OL threshold, a parameter to describe the OL efficiency, is defined as the energy where the transmittance drops to 50% of the initial linear value. It can be seen that BNNT presents the best OL performance, much better than MWCNT. BNNT exhibits the lowest OL threshold and the overall highest attenuation in the NL regime, followed by MWCNT, BNNP, and BNNS. The OL threshold is located at 2 μJ for BNNT, 50 μJ for BNNS, 22 μJ for BNNP, and 4 μJ for MWCNT. BNNT reaches an optical density (OD) due to OL behaviour of 2.1, BNNS of 1.3, BNNP of 1.5, and MWCNT of 1.7 at 2 mJ input energy. The impinging energy on detector D3 is 4.7 μJ, 40.5 μJ, 26.1 μJ, and 7.0 μJ for BNNT, BNNS, BNNP, and MWCNT, respectively. All of the determined values are given in Table 1.

Relevant NL scattering occurs in all samples. BNNT shows the largest NL scattering at 30° in the forward direction, followed by BNNP, BNNS, and MWCNT. NL scattering starts at around 22 μJ for MWCNT and around 10 μJ for all BN samples. According to Mie theory, no relevant scattering can be achieved by particles much smaller than the wavelength of the incident radiation. Scattering becomes effective when further, significantly larger scattering centres are formed by interaction of the laser radiation with the nanoparticles. Thus, NL scattering is always an induced process, initiated by absorption (see Section 2.1).

To analyse the contribution of NL scattering in the OL process in more detail, the angular distributions of the scattered energy at three incident energy levels were measured (Figure 8, right-hand side). The utilised energies were 25 μJ, which is close to the OL threshold, 250 μJ and 600 μJ in the NL region. The scattering characteristics of all samples show an asymmetric dumbbell shape. In the case of BNNP, most of the energy scattered in the forward direction is emitted over a wide angular range of up to 68° at every measured input energy. The angle where the scattered energy drops to 50% of the maximum value was used to define the angular range. In addition, a large amount of radiation is scattered in the backward direction. The scattering angles of BNNS and BNNT are smaller. In the case of BNNS, the majority is radiated up to 58°, 55°, and 55°, the angular ranges of BNNT are 60°, 58°, and 57° at 25 μJ, 250 μJ, and 600 μJ, respectively. The ratios of forward to backward scattering are 3.1, 3.1, and 3.3 for BNNT, 2.9, 3.2, and 3.0 for BNNS, and 2.1, 2.2, and 2.0 for BNNP at 25 μJ, 250 μJ, and 600 μJ, respectively.

**Figure 9:**
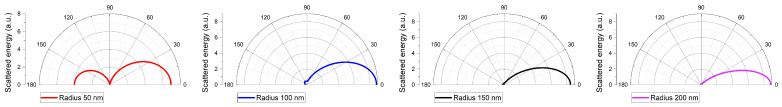
Simulations of angle-dependent Mie scattering with scattering centres of different sizes, with a fixed refractive index of n = 2 + 1i for all simulations.

Belousova et al. developed a theoretical model based on Mie theory in order to qualitatively describe the OL of carbon nanoparticles in dispersion under nanosecond laser irradiation [30,31]. In their model, the energy of the laser radiation is sufficiently high to heat the particles above the boiling point of the solvent, but not that high that the particles themselves evaporate. To illustrate the influence of the scattering centre size on Mie scattering, simulations of angle-dependent Mie scattering with scattering centres of different sizes (50 nm, 100 nm, 150 nm, and 200 nm) were performed (Figure 9). The refractive index was chosen to be n = 2 + 1i, since a refractive index around this value has been obtained for colloidal carbon in the visible region [51]. The simulations were generated using the software MiePlot. The size of the scattering centres influences the ratio of forward to backward scattering. The larger the scattering centre, the more is scattered in the forward direction—the ratio of forward to backward scattering thus increases significantly. It also has an influence on the shape of the scattering characteristics. The larger the scattering centres, the smaller the scattering angles. When comparing our measurements with the simulations, the differences between them are immediately obvious: neither backward scattering nor scattering angles decrease significantly in the measured curves. The angular range and the ratio of forward to backward scattering remain almost constant.

At high laser intensities, a laser-induced plasma can arise in the focal spot. If electrons experience a change in momentum due to interactions with ions or atoms in the plasma, bremsstrahlung is emitted (free–free transition); if an electron recombines with an ion, recombination radiation is generated (free–bound transition). In both cases continuum radiation is emitted [52]. To measure the plasma emission, a notch filter (Edmund Optics, OD > 6, centre wavelength 532 nm, FWHM 17 nm) was placed in front of detector D2. The radiation emitted by the plasma can then be detected since the laser wavelength, and thus the scattered signal at 532 nm, is blocked. Plasma emission arises at around 50 μJ in the BN samples and around 100 μJ in MWCNT. In the case of the scattering measurements, the portion of plasma radiation around 532 nm is also measured by detector D2 when using a bandpass filter. Therefore, the measured radiation is composed of the plasma emission and the NL scattering of the laser beam. The scattering characteristics measured in the high energy range when a plasma arises thus can no longer be described by Mie scattering solely.

**Figure 10:**
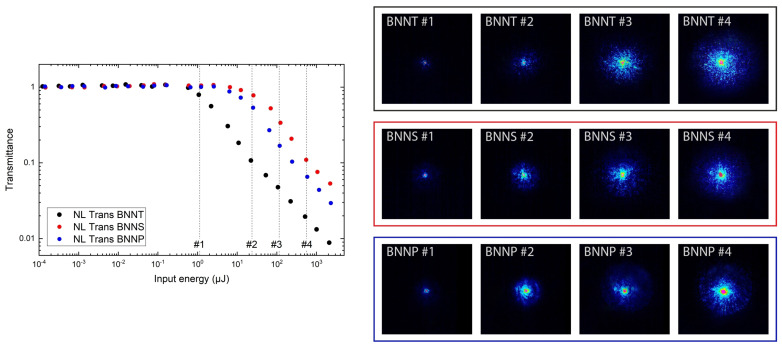
Normalised NL transmittance as a function of the incident pulse energy of BNNT, BNNS, and BNNP. The energy values chosen to characterise the transmitted laser beam are marked with #1 to #4 (**left**). Beam characteristics at four different input energies (#1 to #4) for BNNT, BNNS, and BNNP samples (**right**).

The beam characteristics of the transmitted laser pulses at four different input energies for BNNT, BNNS, and BNNP are shown in Figure 10, on the right. The chosen energy values #1 to #4 are marked in the plot of the normalised NL transmittance. The first energy value, around 1 μJ (#1), is located around the OL threshold for BNNT and in the linear regime for BNNS and BNNP. It can be seen that the laser beam is not affected in any of the samples. At an input energy of 25 μJ (#2), a slight broadening of the laser beam can be observed, this value is in the NL region for all samples. At 115 μJ (#3) and 600 μJ (#4), a significant broadening occurs, with this effect being most pronounced for BNNT.

From these results, which cover the beam characteristics in the forward direction, and from the measurements of the angular distribution, it becomes apparent that NL scattering dominates the OL performance of all measured BN nanomaterials. The laser radiation is dispersed into a larger angular range, which reduces the resulting energy density of the incident beam. When having a closer look at the centre of the laser beam, it can be seen that the energy density at the centre is significantly lower in the case of BNNT compared to BNNS and BNNP. To strongly reduce the energy density at the centre is a key factor for laser protection applications in NL scattering samples.

## 5. Conclusions

In summary, we studied the OL properties of dispersed boron nitride nanotubes, nanoparticles, and nanoplatelets regarding nanosecond laser pulses at a wavelength of 532 nm. To the best of our knowledge, this is the first detailed and comparative analysis on the NL response of these nanomaterials. To evaluate their OL performance, MWCNT is used as a reference. BNNT was found to exhibit a large OL effect, much stronger than the benchmark material MWCNT.

We investigated the beam characteristics of the transmitted and the angular distribution of the scattered signals. Thus, we were able to characterise the entire transmittance and scattering behaviour with our measurements. The angular distributions of scattered light at different energy levels were measured to elucidate the contribution of NL scattering within the NL attenuation process. The scattering characteristics of all samples show an asymmetric dumbbell shape. BNNT shows the largest NL scattering at 30° in the forward direction, followed by BNNP, BNNS, and MWCNT. A theoretical model based on Mie theory is often used to describe NL scattering under nanosecond laser irradiation. Our results revealed that the scattering characteristics measured in the high energy range when a plasma arises can no longer be described by Mie scattering solely. In addition, the beam characteristics of the transmitted laser pulses at different input energies were recorded and analysed with the help of a beam profiling camera. The laser radiation is dispersed into a larger angular range, which reduces the resulting intensity of the incident beam.

Our investigations showed that NL scattering dominates the OL performance of all measured BN nanomaterials. Due to its large OL effect, in particular BNNT is a very good candidate for laser protection applications.

## Figures and Tables

**Table 1:** Optical limiting parameters of BNNT, BNNP, BNNS, and MWCNT (OL threshold, OD at 2 mJ, transmitted energy within 1.5 mrad (E_t_) and scattering (30°) at 2 mJ input energy).

Sample	OL Threshold (μJ)	OD at 2 mJ	E_t_ (μJ) at 2 mJ	Scattering 30° (a.u.) at 2 mJ
BNNT	2	2.1	4.7	317
BNNP	22	1.5	26.1	278
BNNS	50	1.3	40.5	230
MWCNT	4	1.7	7.0	202

## Data Availability

The data presented in this study are available on request from the corresponding author.

## Abbreviations

The following abbreviations are used in this manuscript:

BN	Boron nitride
BNNP	Boron nitride nanoparticles
BNNS	Boron nitride nanoplatelets
BNNT	Boron nitride nanotubes
ESA	Excited-state absorption
FCA	Free-carrier absorption
MPA	Multi-photon absorption
MWCNT	Multi-walled carbon nanotubes
NL	Nonlinear
NLO	Nonlinear optical
OL	Optical limiting
PVP	Polyvinylpyrrolidone
1PA	One-photon absorption
2PA	Two-photon absorption
RSA	Reverse saturable absorption
3PA	Three-photon absorption
VIS	visible
